# Emergency department volume metrics and the association with falls

**DOI:** 10.1016/j.acepjo.2025.100074

**Published:** 2025-03-03

**Authors:** Jesse A. Schacht, Sydney Mulqueen, Alyssa Mangino, Peter S. Antkowiak, Ryan C. Burke, Bryan A. Stenson, David T. Chiu

**Affiliations:** Department of Emergency Medicine, Beth Israel Deaconess Medical Center, Boston, Massachusetts, USA

**Keywords:** crowding, boarding, fall, ED volume, fall prevention, patient safety, emergency department operations

## Abstract

**Objectives:**

Falls in hospitals are common and contribute to morbidity and resource utilization, with limited data from emergency departments (EDs). ED volumes are high with significant levels of crowding and inpatient boarders. The relationship between ED volume and falls is not yet established. We aimed to characterize the population of ED falls, and evaluate their relationship between crowding and boarding.

**Methods:**

Retrospective cohort study from an academic ED in Boston, MA, USA, with 55,000 annual visits between April 1, 2016, and October 31, 2023. The primary outcome was the presence of a fall during the ED stay. We captured age, sex, emergency severity index (ESI) score, arrival times, chief complaint, and total ED volume and borders at the time of arrival. Bivariate associations between fall status and volume metrics among other covariates were calculated using chi-squared and 2-sample t-tests. A logistic regression model was built for each volume metric.

**Results:**

There were 393,876 total visits and 456 documented falls. The fall rate was 0.12%. With an adjusted odds ratio (OR) of 1.013 (95% CI, 1.004-1.022), the number of patients in the ED at the time of the fall was statistically significantly related to falls.

**Conclusion:**

Increased ED volume at the time of patient arrival—a measure of crowding— is associated with an increased risk of falls for ED patients. Boarding does not have an association, even when controlling for demographics and chief complaints intuitively associated with falls. This information may help inform departments when to enact additional preventative measures.

**The Bottom Line**

This study aimed to characterize the emergency department population experiencing falls and assess the correlation between falls, total departmental volume, and the number of boarders. We controlled for emergency severity index, arrival year, age, gender, race, and primary language. Visits with falls were more likely to be older, male, and have a chief complaint related to alcohol or previous falls. Total volume at the time of patient arrival was associated with an increased risk of falls (odds ratio [OR], 1.013; 95% CI,1.004-1.022). This association was not present for the number of boarding patients (OR, 1.011; 95% CI, 0.995-1.028]).

## 1 Introduction

### 1.1 Background

Falls in health care settings are common and contribute significantly to morbidity, length of stay, cost, and resource utilization.^1^ Nearly 1 million falls occur annually across all health care settings, a third of which result in injury.^2^^,^^3^ Geriatric patients suffer more serious consequences from falls than others; falls in this population are now considered a major traumatic mechanism.^4^ As a nation, we spend around $28 billion on fall-related injuries and hospitalizations. Importantly, the Centers for Medicare and Medicaid Services do not reimburse hospitals for costs associated with in-hospital falls.^3^ Despite efforts at standardizing fall-risk evaluation and prevention, they still occur relatively frequently, and prevention of falls remains a challenge for many health care institutions.

There is limited available data regarding falls that specifically occur in the emergency department (ED), even though the ED is a setting that cares for patients with various medical problems intuitively associated with high-fall risk.^5^ One retrospective study from 2016 in Australia identified commonalities between inpatient falls and ED falls, including patient-specific factors and medications.^5^ The data regarding which ED patients are at highest risk for falls and the characteristics of the department at the time of the falls are lacking.

### 1.2 Importance

Contemporary EDs now face unprecedented levels of inpatient crowding and boarding.^6^ As ED volumes and crowding continue to increase, it is imperative to understand how these factors contribute to fall risk in the ED. As published in numerous studies, the effects of ED crowding on patient care and satisfaction are substantial. Crowding is associated with negative patient outcomes.^6^, ^7^, ^8^ The relationship between ED volume metrics and falls in the ED is not yet established in the literature. More data regarding the characteristics of patients who fall in the ED, and the relation of falls to ED volume metrics is warranted.

### 1.3 Goals of This Investigation

In this study, we characterized the population that experienced falls while in the ED and investigated the relationship between ED volume metrics and the likelihood of a fall. Understanding departmental and patient characteristics associated with falls can improve processes aimed at reducing both harm and costs associated with falls.

## 2 Methods

### 2.1 Design and Setting

This was a retrospective cohort study using existing electronic health record data on visits to a single ED from April 1, 2016, until October 31, 2023. The study institution is a tertiary academic center with around 55,000 annual visits. The average daily patient visit count is around 150 visits per day. There are 63 licensed beds available, in addition to up to 12 hallway beds. There is an 11-bed medical observation unit within the ED as well as a 5-bed behavioral health unit. Patients in the ED observation or behavioral units are not boarders—they are observation class patients.

There is a hospital-level “Code Help” policy to mitigate crowding and ensure the ED can maintain the ability to manage new patients. This was authored by hospital administrators to activate surge spaces for clinically appropriate patients. This goes into effect when the ED has ≥63 registered patients and ≥10 boarding patients present for 2 consecutive hours.

At the study institution, fall-risk patients are not necessarily monitored in a 1:1 manner. The individuals responsible for watching include nurses, technicians, and designated sitters.

Ideal nursing staffing at the study institution on weekdays is 24 nurses from 11 AM to 11 PM, 21 nurses from 11 PM to 3 AM, and 18 nurses from 11 PM to 11 AM. Ratios ideally range from 1 nurse to every 3 patients in the most acute zones to 1 nurse to every 6 patients in the low acuity observation zone. Ideal technician staffing on weekdays is 16 to 18 from 11 AM to 11 PM and 12 to 14 from 11 PM to 11 AM.

### 2.2 Selection of Subjects

We included all patients aged ≥18 years who visited the ED and were roomed in the ED.

### 2.3 Interventions

No interventions were performed as this was a retrospective cohort study.

### 2.4 Measures and Outcomes

The primary outcome measure was the presence of a fall during the ED stay. All falls are reportable events, and this information (ie name, date, severity level, and circumstances around the fall) is entered into a hospital-wide reporting system by the nurse. All ED falls during the study period were collected from this database and mapped to the ED encounter when they occurred.

The primary independent variable of interest was ED volume. This was defined using 2 separate metrics, both captured at the time of the patient’s arrival at the ED. The first volume metric was total census, defined as the number of patients in the ED. The second metric was boarding, defined as the number of patients admitted to the hospital, though still physically occupying an ED bed space while awaiting an inpatient bed for ≥2 hours.

Additional covariates, chosen a priori, collected for each ED visit were emergency severity index (ESI) score, year of arrival, age, gender, race, and whether or not English was the patient’s primary language. We also assessed each chief complaint for the presence of the word “fall,” or any relation to alcohol intoxication, as these populations anecdotally experience more falls. Separate categoric variables were created to indicate whether or not the visit included a chief complaint of fall or a chief complaint of alcohol intoxication.

### 2.5 Data Analysis

Data were summarized overall and stratified by fall status. Bivariate associations between fall status and the volume metrics as well as covariates were calculated with a chi-square test for categoric variables and a 2-sample t-test for continuous variables. A *P*-value of 0.05 was considered significant. To assess the relationship between falls and ED volume, adjusting for the covariates listed above, a logistic regression model was built separately for each volume metric. The unit of analysis was a visit. Both ESI score and age were kept continuous in the models, and no transformations were made. Adjusted odds ratios (ORs) and 95% CIs are reported. Data were analyzed using SAS version 9.4.

## 3 Results

There were 393,876 visits included in the study. The total number of falls occurring in the ED was 456, with a fall rate of 0.12%.

Demographic information is summarized in Table 1. The study population was predominantly female (53.7%), White (55.7%), and English-speaking (87.8%). The average age was 54 years; 6.6% came in with a fall chief complaint and 3% came in with an alcohol-related chief complaint. Visits with a fall were more likely to be male and White (*P* = .0151 and *P* < .0001, respectively). Visits with a fall were more likely to have a fall or alcohol-related chief complaint in the ED (*P* < .0001 for both). The patient population that fell had a higher mean age (59 vs 54 years, *P* < .0001) and lower mean ESI score (2.4 vs 2.6, *P* < .0001).

**Table 1:** Demographic characteristics and visit information of emergency department visits overall and stratified by fall status.

Characteristic	Overall (N = 393,876)	Fall (n = 456)	No Fall (n = 393,420)	*P*-value
Sex				.0151
Female	211,501 (53.7%)	219 (48.0%)	211,282 (53.7%)	
Male	182,375 (46.3%)	237 (52.0%)	182,138 (46.3%)	
Race				< .0001
Asian	20,214 (5.1%)	16 (3.5%)	20,198 (5.1%)	
Black	88,957 (22.6%)	74 (16.2%)	89,883 (22.6%)	
Hispanic	34,688 (8.8%)	27 (5.9%)	34,661 (8.8%)	
Other	23,540 (6.0%)	19 (4.2%)	23,521 (6.0%)	
Unknown	6,970 (1.8%)	4 (0.9%)	6,966 (1.8%)	
White	219,507 (55.7%)	316 (69.3%)	219,191 (55.7%)	
Language				.0371
English	345,890 (87.8%)	415 (91.0%)	345,475 (87.8%)	
Not English	47,986 (12.2%)	41 (9.0%)	47,945 (12.2%)	
Fall chief complaint				< .0001
No	367,904 (93.4%)	362 (79.4%)	367,542 (93.4%)	
Yes	25,972 (6.6%)	94 (20.6%)	25,878 (6.6%)	
Alcohol intoxication chief complaint				< .0001
No	382,215 (97.0%)	410 (89.9%)	381,805 (97.0%)	
Yes	11,661 (3.0%)	46 (10.1%)	11,615 (3.0%)	
Age (y), mean (±SD)	53.9 (20.6)	59.2 (20.3)	53.9 (20.6)	< .0001
ESI, mean (±SD)	2.6 (0.7)	2.4 (0.6)	2.6 (0.7)	< .0001
No. in the ED, mean (±SD)	53.8 (11.4)	55.6 (10.1)	53.8 (11.4)	.0002
No. of boarders, (±SD)	6.1 (5.6)	6.5 (5.6)	6.1 (5.6)	.118

ESI, emergency severity index.

The mean number of patients in the department at the time of ED arrival was 54 and the mean number of boarders was 6. The mean number of patients in the department at the time of arrival for visits with a fall was higher than for visits without a fall (55.6 vs 53.8, *P* = .0002). The mean number of borders at the time of arrival for visits with a fall was not different than the mean number for visits without a fall (6.5 vs 6.1, *P* = .118).

The results of the multivariate analyses of the 2 crowding factors are shown in Tables 2 and 3. For each additional patient in the ED at the time of arrival, the odds of a fall significantly increased by 1.013 (95% CI, 1.004-1.022), after adjusting for the measured confounders (Table 2). For each additional boarder in the ED at the time of arrival, the odds of a fall did not significantly increase (OR, 1.011; 95% CI, 0.995-1.028) after adjusting for the measured confounders.

**Table 2:** Logistic regression results of the relationship between total ED volume and fall risk.

Variable	Adjusted odds ratio	95% Lower CI	95% Upper CI
Total ED Volume	1.013	1.004	1.022
Year	1.054	1.011	1.098
ESI score	0.808	0.701	0.931
Age (y)	1.008	1.003	1.013
Sex F vs M	0.876	0.726	1.057
Chief complaint of fall	2.98	2.35	3.777
Chief complaint of alcohol intoxication	3.811	2.773	5.237

ED, emergency department; ESI, emergency severity index; F, female; M, male.

Additional covariates included in the model are race/ethnicity and language.

**Table 3:** Logistic regressions results of the relationship between emergency department boarders and fall risk.

Variable	Adjusted odds ratio	95% Lower CI	95% Upper CI
Total boarder patients	1.011	0.995	1.028
Year	1.059	1.016	1.105
ESI score	0.803	0.697	0.925
Age (y)	1.008	1.003	1.013
Sex F vs M	0.878	0.728	1.059
The chief complaint of fall	2.964	2.338	3.758
Chief complaint of alcohol intoxication	3.868	2.814	5.317

ESI, emergency severity index; F, female; M, male.

Additional covariates included in the model: are race/ethnicity and language.

### 3.1 Limitations

This study was conducted at a single institution. Despite the large data set, specific practices within the institution may preclude generalizability.

The data were generated by evaluating the conditions of the ED at the time of patient arrival. The ED is a dynamic environment with continually changing patient volumes and conditions. A patient may have arrived at the ED during a period of relatively few total patients. However, during their ED stay, the number of patients may have significantly increased. The fall risk at the time of arrival may have been minimal, but factors not measured in this study may have contributed to a dynamic fall risk for individual patients. This data do not characterize the conditions of the ED at the time of each patient fall. Although the presence of a fall was documented with free text details, the exact minute and hour of the fall were not readily available in the data, driving us to choose volume metrics at the time of arrival.

Operations, nursing, and bed management protocols in the ED at the study institution include activation of “Code Help” when the ED volume crosses a pre-defined threshold. There are no fall-specific measures that occur with the activation of this code. The downstream effects of activating Code Help are mainly to increase the ability to move admitted patients to their respective inpatient units, as well as increase available nursing staff by calling in on-call nurses and allowing for temporary hallway space to be established in inpatient units. It is possible that during periods of significant crowding and/or boarding, Code Help was activated and influenced the fall risk for patients in the ED during that time.

Additionally, certain patients are inherently more likely to fall—most notably those with prior falls or those who are intoxicated. The relative weight of the conditions of the department in terms of their contribution to fall risk was not measured. Patient-specific characteristics, including age, gender, comorbidities, chief complaint, medications associated with falls, and previous falls, among others, may outweigh the conditions of the department in terms of their contribution to fall risk. Additionally, we were unable to assess whether the fall occurred in a patient room in a hallway bed or in a standard ED room.

The details of each fall that occurred in the ED during the study period were not analyzed. Whether an injury was sustained, what the circumstances of each fall were, or whether polypharmacy, medications, or intoxication played a role was not elucidated. The activity at the time of the fall was also not collected, and thus, although it is possible that most falls occurred during toileting, for example, we cannot comment.

## 4 Discussion

The patients more likely to sustain a fall were male, older, and presenting to the ED with chief complaints of either falls or alcohol intoxication. In the study cohort, there was a statistically significant relationship between falls and chief complaints of fall or alcohol intoxication. These results are intuitive, as these patients would be deemed a high-fall risk and are likely to experience falls irrespective of being present in the ED.

Our study demonstrated higher odds of falling as the total ED volume at the time of arrival increased. Despite appearing to be only slightly elevated, the ORs for falling in a crowded ED remains clinically significant. Applying a small increase in risk to a large study population results in a significant number of patients experiencing falls. Any preventable fall while in the ED or admitted is taken seriously by the hospital.

The association between falls and ED volume is likely multifactorial. As the ED and waiting rooms accumulate patients, it is likely harder to observe and prevent falls in high-risk individuals. To prevent future falls in the ED, it may be possible to establish ED volume thresholds beyond which certain measures are taken to heighten departmental awareness of falls. Preventative measures for falls generally consist of patient sitters, bed alarms and sensors, and nursing risk assessment tools.

Boarding admitted patients in the ED while awaiting an inpatient bed is becoming increasingly more common. ED boarding negatively impacts ED throughput, patient safety and outcome measures, and physician-specific concerns, including job satisfaction. However, our study did not find a statistically significant relationship between ED boarding at the time of arrival and the likelihood of falling. During the study period, there did not appear to be large boarding numbers, with a mean of 6.1 boarders present at the time of arrival. Current boarding of inpatients anecdotally exceeds the level during the study period and may prove to be associated with increased fall risk in future studies.

Where there are 10 boarders present for 2 consecutive hours (with a total census of over 63), our hospital activates surge capacity to offload some volume from the ED via a Code Help policy. However, there are still many barriers to getting these patients admitted efficiently, and it can take many hours for the volume to fall below those thresholds.

Although sparse data regarding ED-specific falls is published, 1 retrospective study identified commonalities between risk factors for inpatient falls and those that occur in a single tertiary ED.^5^ This study adds to that literature and also uncovers an association with crowding. As crowding is associated with increased morbidity and mortality,^7^ it is imperative to understand its impact on all aspects of patient safety. Crowding leads to assessment delays and increased exposure to errors,^6^^,^^8^ so an additional association with falls is worthwhile to recognize.

Recognizing this association, there may be increased benefits in examining fall prevention strategies and their optimal implementation. The American College of Emergency Physicians (ACEP) policy statement for Geriatric Emergency Department Guidelines details features of EDs that may assist in fall prevention in the geriatric population. These guidelines include specific furniture improvements, particularly chairs/recliners, as well as those with sturdy armrests. The ACEP policy notes that falls tend to occur while patients are attempting to get out of bed unsupervised or unassisted. Interestingly, bedrails are not associated with decreased falls and may worsen their severity. This contradicts policies that recommend raising bedrails, a suggestion we did not address in our study but which deserves further investigation. The ACEP policy statement also suggests formal physical therapy evaluation of patients identified as fall risks.^4^ Although this policy primarily targets the geriatric population, certain aspects are also relevant to the ED population as a whole. The study institution is not currently designated a Geriatric Emergency Department. Additionally, the efficacy of fall prevention measures was analyzed in a meta-analysis in *Age Ageing* in 2022. Their data suggests the only intervention with a reduced OR of falls was patient and family education. Bed alarms, sensors, and risk assessment tools did not significantly reduce falls in their study.^1^ However, future studies looking at compliance with these suggestions at varying levels of crowding—when falls are more likely—may be insightful.

Despite the lack of efficacious and standardized fall prevention measures, the relationship between crowding and falls should not go unnoticed. ED clinicians should have heightened awareness of fall risk as emergency departments become more crowded. Implementation of facility-specific fall prevention measures beyond a certain ED volume should be considered.

## Author Contributions

The study was designed and devised primarily by JAS, BAS and DTC. Data were collected and organized primarily by BAS. RCB performed statistical analysis and assisted in drafting the methods section. SM and AM assisted in editing the manuscript as well as presented the preliminary data at SAEM, 2024. JAS wrote and edited the manuscript, assisted in the study scope and design, assisted in the analysis of data, performed the literature review, and wrote the preliminary abstract for the SAEM presentation. All authors contributed substantially to its revision.

## Funding and Support

By *JACEP Open* policy, all authors are required to disclose any and all commercial, financial, and other relationships in any way related to the subject of this article as per ICMJE conflict of interest guidelines (see www.icmje.org). The authors have stated that no such relationships exist.

## Conflict of Interest

All authors have affirmed they have no conflicts of interest to declare.
